# Supplementary material for: The role of individual and community empowerment as drivers of contraceptive use among reproductive aged women in Bangladesh: Insights from multilevel analysis using BDHS 2022
Source: PLoS One. 2026 Jan 21;21(1):e0337499. doi: 10.1371/journal.pone.0337499 (PMC12822922; doi:10.1371/journal.pone.0337499)
Supplement: Attachment — Submitted filename: Response to Reviewers.docx [file pone.0337499.s001.docx]

**Manuscript ID**: PONE-D-25-12603
**Title**: *The Role of Individual and Community Empowerment as Drivers of Contraceptive Use among Reproductive Aged Women in Bangladesh*

Dear Editor and Reviewer,

We sincerely thank you for your thoughtful and constructive feedback on our manuscript. We appreciate the opportunity to revise and resubmit our work. Below, we respond to each point raised and describe how we have addressed them in the revised manuscript.

**Reviewer Comments and Author Responses**

**1. Conceptual and Theoretical Limitations**

**Comment 1.1**: The socio-ecological theory was mentioned but not elaborated.
**Response**: We appreciate the reviewer’s observation. In response, we have expanded the Introduction (lines 84–90), Methods (lines 115–121), and Discussion (lines 252–253) sections of the revised manuscript to more clearly articulate how the socio-ecological theory informs the conceptual framework and analytical approach of our study.

**Comment 1.2**: Lack of clear differentiation between individual and community empowerment.
**Response**: Thank you for the insightful comment. We have now provided clearer and more precise definitions to distinguish between individual and community-level empowerment in the Methods section (lines 115–121) of the revised manuscript.

**2. Methodological Weaknesses**

**Comment 2.1**: Dataset limitations like self-reporting bias not discussed.
**Response**: We have acknowledged potential biases in the ‘Limitations and Future Research’ section, specifically noting social desirability bias related to self-reported contraceptive use in BDHS.

**Comment 2.2**: Lack of causal mechanism analysis.
**Response**: We appreciate this important observation. In the revised manuscript, we have explicitly acknowledged the limitation regarding causal inference in the “Limitations and Future Research” section. We have suggested that future studies use advanced causal modeling techniques (e.g., mediation or moderation analysis) to explore potential causal mechanisms.

**Comment 2.3**: Socio-economic variables not explored in depth.
**Response**: Given that all eight socio-economic factors considered in this study were significantly associated with contraceptive use in our analysis (Table 3), and that community empowerment also had a strong, independent effect; it is plausible that these socio-economic factors may enhance or condition the impact of community-level empowerment. Although we did not formally test for interaction effects, future research could explore whether these factors moderate the relationship between empowerment and contraceptive use.

**3. Interpretation and Policy Implications**

**Comment 3.1**: Policy implications are general.
**Response**: We appreciate this suggestion. To address it, we have revised the ‘Implications for Policy and Practice’ section by adding specific examples of community-based interventions, such as women’s support groups, community health volunteers, and engagement of local leaders, which can strengthen community empowerment and enhance contraceptive uptake. Additionally, we have updated the ‘Abstract’ (line 37-40) to reflect these specific recommendations in a more concise and actionable manner.

**Comment 3.2**: Need for contextualization within Bangladeshi sociocultural norms.
**Response**: We have incorporated additional contextual information on gender norms, religious practices, and the influence of patriarchy in Bangladesh. These revisions have been added to the Introduction (lines 63–70) of the revised manuscript to strengthen the socio-cultural framing of the study.

**4. Writing and Structure**

**Comment 4.1**: Repetitiveness and vague claims.
**Response**: We appreciate the reviewer’s feedback regarding repetitiveness and vague claims. In response, we have revised the Introduction section to improve clarity and conciseness. Specifically, we deleted the sentence referring to “notable investment” in family planning and women’s empowerment, as it was vague and not supported by specific evidence. Additionally, we streamlined and consolidated several overlapping statements discussing the influence of community-level empowerment on contraceptive use. These edits reduce redundancy and enhance the overall focus of the introduction by clearly articulating the study’s objective and contextual framework.

**5. Literature and Framework**

**Comment 5.1**: Literature review is descriptive, not analytical.
**Response**: Thank you for this insightful comment. In response, we have revised the literature review in the Introduction (lines 53–55) to adopt a more analytical tone. The revised section now critically engages with prior findings by highlighting inconsistencies—for example, how education alone may not explain contraceptive use disparities in rural or conservative contexts—and by identifying gaps that our study seeks to address. These changes provide a stronger rationale for examining both individual- and community-level empowerment and enhance the conceptual framing of our research.

**Comment 5.2**: Terms like “community empowerment” need clearer definitions.
**Response**: We now define such terms clearly in the methods section and operationalize how they were measured.

**6. Statistical and Model Concerns**

**Comment 6.1**: Justification for PCA vs other factor analysis methods missing.
**Response**: We appreciate this comment. We have now clarified in the manuscript (line 152-158) that Principal Component Analysis (PCA) was chosen because it is a standard method used in DHS analyses, particularly for constructing composite indices such as the wealth index. Our application of PCA to empowerment follows the same rationale and methodology.

**Comment 6.2**: Explanation of model assumptions and diagnostics needed.
**Response:** We would like to respectfully note that we have already addressed the key model diagnostics in the originally submitted manuscript. Specifically:

1. The use of intra-cluster correlation (ICC) and the justification for a multilevel logistic regression model are discussed at lines 168–169.
2. The assessment of multicollinearity using Variance Inflation Factor (VIF) is stated at line 177-178.

If the reviewer requires more detailed explanation—such as specific ICC or AIC values, or expanded discussion of additional model assumptions—we would be happy to incorporate those upon request.

**7. Ethical Considerations and Data Availability**

**Comment 7.1**: Ethics section could address informed consent, anonymization, and researcher bias.
**Response**: We revised the ethics section to include explicit mention of informed consent, anonymized data, and our use of publicly available secondary data, reducing risk of bias.

**Response to the Editor**

**PLOS Formatting and Journal Requirements**

**Response**: We confirm that no funding information is included in the manuscript, as required. In the online submission form, we want to retain the previous statement: “The authors received no specific funding for this work.”

In addition, we have double-checked all references for formatting consistency and citation integrity. No retracted articles were found in the reference list.

As part of this revision, we have shifted the previous reference 5 to position 3, and have added a new reference (now listed as reference 17) to appropriately cite the use of the chi-square test and multilevel logistic regression model.
