# Supplementary material for: The role of individual and community empowerment as drivers of contraceptive use among reproductive aged women in Bangladesh: Insights from multilevel analysis using BDHS 2022
Source: PLoS One. 2026 Jan 21;21(1):e0337499. doi: 10.1371/journal.pone.0337499 (PMC12822922; doi:10.1371/journal.pone.0337499)
Supplement: Attachment — Submitted filename: Response to Reviewer .docx [file pone.0337499.s002.docx]

**Response to Reviewer Comments**

**Manuscript ID:** PONE-D-25-12603R1
**Title:** *The Role of Individual and Community Empowerment as Drivers of Contraceptive Use among Reproductive Aged Women in Bangladesh*

We sincerely thank the reviewer and the editor for their valuable feedback and constructive suggestions. We have carefully revised the manuscript to address all points raised. Below is a summary of the major revisions made:

**1. Clarity and Conciseness:**
The manuscript was detailed, but sections such as the *Introduction* and *Discussion* have now been revised for greater clarity and brevity. We summarized background information more succinctly to engage readers quickly and improve flow. Several lines in the *Introduction* and *Discussion* were rewritten to make the text more concise and focused.

**2. Data Presentation:**
The reviewers suggested improving the readability of the Tables. Significant *p*-values are now highlighted in bold for quick reference.

**3. Statistical Analysis:**
While the original methods were robust, additional visualization was recommended to better communicate key findings. We have added a new heatmap figure to complement the tabular results and included corresponding text in the *Results* section describing and interpreting the figure.

**4. Discussion Depth:**
Reviewer recommended a deeper discussion on the cultural and social factors that might explain the inverse relationship between wealth and contraceptive use. We added new lines at the end of the *Discussion* section elaborating on the potential cultural and social influences underlying this unexpected finding.

**5. Limitations and Future Research:**
The reviewers noted that the *Limitations* section could better articulate how the cross-sectional design affects causal interpretation and provide more specific guidance for future research. We expanded the *Limitations* section to clarify the implications of the study’s cross-sectional nature on causal inference.
Additionally, we updated the *Future Research* section to include specific suggestions, such as the use of longitudinal data and mixed-method approaches, along with potential research questions to explore the empowerment–contraceptive use nexus in more depth.
