# Supplementary material for: The role of individual and community empowerment as drivers of contraceptive use among reproductive aged women in Bangladesh: Insights from multilevel analysis using BDHS 2022
Source: PLoS One. 2026 Jan 21;21(1):e0337499. doi: 10.1371/journal.pone.0337499 (PMC12822922; doi:10.1371/journal.pone.0337499)
Supplement: Attachment — Submitted filename: Response_to_Reviewers_auresp_3.docx [file pone.0337499.s003.docx]

**Response to Reviewers**

PONE-D-25-12603R2

*The Role of Individual and Community Empowerment as Drivers of Contraceptive Use among Reproductive Aged Women in Bangladesh*

We thank the editor and the reviewers for their careful reading of our manuscript and their constructive comments. We appreciate the detailed feedback and have revised the manuscript accordingly. Below we provide a point-by-point response.

**Reviewer #1**

We sincerely thank Reviewer #1 for their careful reading and positive feedback on our previous revisions. We appreciate the recognition of the improvements made in clarity, data presentation, statistical visualization, discussion depth, and limitations.

**Reviewer #2**

We sincerely thank Reviewer #2 for the detailed and constructive feedback. All suggested revisions have been incorporated, significantly improving the manuscript’s clarity, rigor, and relevance.

**1. Title, Abstract, and Framing**
**Comment:** The title could better reflect the multilevel design; abstract should specify statistical methods and marginal association of individual empowerment; framing overemphasizes limited access to family planning.

**Response:** Thank you for these suggestions.

- The title has been revised to:
  *“The role of individual and community empowerment as drivers of contraceptive use among reproductive aged women in Bangladesh: Insights from multilevel analysis using BDHS 2022.”*
- The abstract now explicitly mentions the Mann–Whitney U test, chi-square tests, and multilevel logistic regression, and notes that individual empowerment was only marginally associated (p≈0.10).
- Statements regarding limited access to contraception have been adjusted to avoid overgeneralization.

**2. Introduction and Conceptualization**
**Comment:** Conceptual framework and gap statement needed; some references misaligned.

**Response:** We appreciate these deep findings. In response,

- A concise paragraph has been added at the end of the Introduction to describe the conceptual framework, showing the pathways through which individual and community empowerment may influence contraceptive use.
- The gap statement has been clarified to emphasize the novelty of using BDHS 2022, multilevel community empowerment index, and PCA-based empowerment scores.
- Reference misalignments, including Reference 1, have been corrected and updated.

**3. Methods – Data, Variables, and Analysis**
**Comment:** Clarify exclusion criteria and handling of DHS weights, strata, and clusters; justify collapsing contraceptive use into a binary variable.

**Response:**

- Exclusion criteria are now explicitly described: non-married women, missing contraceptive status, and age restrictions were excluded. DHS weights, strata, and primary sampling units are also described.
- Contraceptive use was dichotomized as “any use” versus non-use, consistent with prior DHS studies, to ensure sufficient statistical power. A supporting reference has been added.

**4. Empowerment Measures**
**Comment:** Missing data handling, PCA details, coding of wife-beating attitudes, and justification for community empowerment construction needed.

**Response:**

- Women with missing responses on any of the nine individual empowerment indicators were excluded prior to PCA.
- Standard PCA procedures were followed; the first principal component was retained and standardized (mean = 0, SD = 1). PCA results are summarized in the Methods without overloading the manuscript as this is not our core analysis.
- Coding of wife-beating attitudes has been clarified and consistently described.
- Community empowerment is calculated as the mean of individual scores per cluster; justification and a reference are now provided in the manuscript.

**5. Control Variables and Model Specification**
**Comment:** Justify age and wealth categorization; provide multilevel model details and measures of between-cluster variation.

**Response:**

- The papers that are referenced in this manuscript used age as categorical variable.
- Wealth index categories are explicitly linked to DHS quintiles, with a supporting reference.
- The statistical analysis section now specifies the use of a random-intercept multilevel logistic regression model, describes model-building strategy (null model, individual- and community-level models, fully adjusted model), and reports ICC, variance of random effects, MOR, and PCV.

**6. Results – Reporting and Interpretation**
**Comment:** Improve table titles/footnotes; include distributions (IQRs) and effect sizes for Mann–Whitney U test. Confidence intervals for odds ratios are missing from Table 3, and range of VIFs should be provided.

**Response:**

- Table titles now clearly describe the sample (currently married women aged 15–49, BDHS 2022).
- Mann–Whitney U test results include medians, IQRs, and effect sizes to reflect practical significance.
- VIF values ranged between 1.1 and 4.5, indicating no multicollinearity concerns; therefore, we did not report them in detail. Additionally, we opted not to include confidence intervals for odds ratios to avoid redundancy, as p-values are already provided. However, if the reviewer deems it necessary, we are happy to include these in a revised version.

**7. Discussion, Policy Implications, and Limitations**
**Comment:** Strengthen linking of findings to socio-ecological theory and Bangladeshi norms; discuss inverse wealth–contraceptive association; provide more specific policy recommendations; expand limitations.

**Response:**

- The inverse association between wealth and contraceptive use is now interpreted considering method mix, fertility preferences, cultural norms, and male decision-making.
- Policy implications have been revised with specific, context-relevant recommendations, including integrating empowerment into community health worker programs, engaging local leaders, and media campaigns targeting social norms.
- Limitations explicitly cover cross-sectional design, residual confounding, measurement limitations of PCA-based empowerment, and potential cluster-level unobserved factors.

**8. Presentation, Consistency, and Referencing**
**Comment:** Correct language issues, harmonize ethics statements, ensure reference accuracy.

**Response:**

- Language editing was performed to remove repeated phrases, correct minor grammatical errors, and improve clarity.
- Ethical statements were harmonized: the study used publicly available, de-identified secondary data; original DHS protocols had IRB approval.
- References have been checked, updated with recent literature from Bangladesh and South Asia, and aligned with in-text claims.

We sincerely thank the reviewers and the editor again for their thoughtful comments and guidance. We are open to adding further clarifications if the reviewers deem necessary.
